# Post-fall sacral swelling

**DOI:** 10.11604/pamj.2025.51.29.48096

**Published:** 2025-06-04

**Authors:** Haruka Hikichi

**Affiliations:** 1 Department of General Internal Medicine and Clinical Laboratory Medicine, Akita University, School of Medicine, Akita, Japan

**Keywords:** Sacral bursitis, traumatic bursitis, sacral swelling

## Image in medicine

A 54-year-old woman presented with a progressively enlarging, painless swelling over the sacral region. She reported repeated falls over the preceding several weeks, attributed to the adverse effects of antidepressant medications. Physical examination revealed a 15-cm, soft, non-tender mass with mild erythema over the sacrum (panel A). The patient was afebrile. Laboratory findings, including white blood cell count, erythrocyte sedimentation rate, and C-reactive protein, were within normal limits. Computed tomography revealed a well-circumscribed, homogeneous, low-density fluid collection in the subcutaneous tissue over the sacrum (panel B). Aspiration yielded hemorrhagic, turbid fluid (panel C). Cytology results were negative for malignancy, and cultures were negative for bacterial growth. A diagnosis of sacral bursitis was made. Bursitis is an inflammation of a synovium-lined bursa, typically occurring in large joints such as the shoulder, knee, hip, and elbow. Sacral bursitis is rare and often misdiagnosed as an abscess, hematoma, or soft tissue tumor. In this case, the absence of systemic symptoms, normal inflammatory markers, and negative cultures helped rule out an infectious etiology. Imaging findings also lacked the features suggestive of septic bursitis, such as peribursal fat stranding and wall thickening. Most cases of non-infectious bursitis can be managed conservatively. This patient was treated with rest, analgesics, and aspiration drainage, with gradual resolution of symptoms.

**Figure 1:**
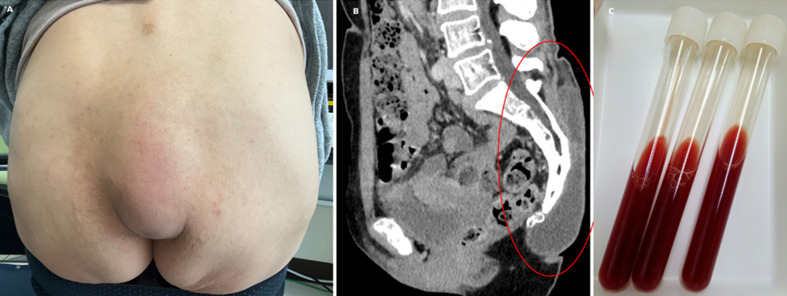
A, B, C) post-fall sacral bursitis presenting as a painless swelling

